# Metabolomic insights into the intricate gut microbial–host interaction in the development of obesity and type 2 diabetes

**DOI:** 10.3389/fmicb.2015.01151

**Published:** 2015-10-27

**Authors:** Magali Palau-Rodriguez, Sara Tulipani, Maria Isabel Queipo-Ortuño, Mireia Urpi-Sarda, Francisco J. Tinahones, Cristina Andres-Lacueva

**Affiliations:** ^1^Biomarkers and Nutrimetabolomic Lab., Nutrition and Food Science Department, XaRTA, INSA, Campus Torribera, Pharmacy Faculty, University of BarcelonaBarcelona, Spain; ^2^Biomedical Research Institute (IBIMA), Service of Endocrinology and Nutrition, Malaga Hospital Complex (Virgen de la Victoria), University of MalagaMalaga, Spain; ^3^CIBER Fisiopatología de la Obesidad y Nutrición (CIBERobn), Instituto de Salud Carlos III (ISCIII)Madrid, Spain

**Keywords:** metabolomics, gut microbiota, obesity, type 2 diabetes, co-metabolism

## Abstract

Gut microbiota has recently been proposed as a crucial environmental factor in the development of metabolic diseases such as obesity and type 2 diabetes, mainly due to its contribution in the modulation of several processes including host energy metabolism, gut epithelial permeability, gut peptide hormone secretion, and host inflammatory state. Since the symbiotic interaction between the gut microbiota and the host is essentially reflected in specific metabolic signatures, much expectation is placed on the application of metabolomic approaches to unveil the key mechanisms linking the gut microbiota composition and activity with disease development. The present review aims to summarize the gut microbial–host co-metabolites identified so far by targeted and untargeted metabolomic studies in humans, in association with impaired glucose homeostasis and/or obesity. An alteration of the co-metabolism of bile acids, branched fatty acids, choline, vitamins (i.e., niacin), purines, and phenolic compounds has been associated so far with the obese or diabese phenotype, in respect to healthy controls. Furthermore, anti-diabetic treatments such as metformin and sulfonylurea have been observed to modulate the gut microbiota or at least their metabolic profiles, thereby potentially affecting insulin resistance through indirect mechanisms still unknown. Despite the scarcity of the metabolomic studies currently available on the microbial–host crosstalk, the data-driven results largely confirmed findings independently obtained from *in vitro* and animal model studies, putting forward the mechanisms underlying the implication of a dysfunctional gut microbiota in the development of metabolic disorders.

## Gut Microbiota And *Diabesity*: Role In Energy Harvest, Gut Barrier Integrity, Endocrine Modulation, And Metabolic Inflammation

Obesity is a complex, multifactorial disease characterized by an excessive accumulation of fat due to an imbalance between energy intake and expenditure. The linear rise in the prevalence of T2D throughout the normal, overweight and obese ranges is so high that the relative risks of diabetes are 40 times higher when BMI increases above 35 kg/m^2^ (Hu et al., 2001; Mokdad et al., 2003; Poirier et al., 2006; World Health Organization [WHO], 2013). The public concern over the obesity epidemic mostly lies in the intimate connection between obesity and T2D (so-called *diabesity*; Astrup and Finer, 2000) and makes the elucidation of mechanisms underlying the co-occurrence of the two diseases a central focus of current biomedical research.

Recently, consideration has started to be given to the gastrointestinal tract as a key point in the development and progression of complex metabolic diseases, since it represents the milieu where interactions between exogenous (i.e., diet, microbiome) and endogenous (i.e., genetic) factors predisposed to disease and the body’s defenses (physical barrier, immune system response) actually take place. Increasing evidence indicates in particular the impact of changes in the composition of the human gut microbiota on host metabolism and a variety of diseases (Bäckhed et al., 2005; Moreno-Indias et al., 2014; Shoaie et al., 2015).

Firmicutes (Gram-positive), Bacteroidetes (Gram-negative) and Actinobacteria (Gram-positive) represent over 90% of the phyla and dominate the gut microbiota (DiBaise et al., 2008), but a relevant change in their relative proportion has been described in obesity and T2D. A favorable prevalence of Firmicutes bacteria toward healthy subjects has been observed in both animal models of obesity (Ley et al., 2005) and human obesity (Ley et al., 2006; Turnbaugh and Gordon, 2009), also reviewed in (Turnbaugh and Gordon, 2009; Sanz et al., 2013; Moreno-Indias et al., 2014), although with some discrepancies among data (Schwiertz et al., 2010). Although the potential impact of specific species on host metabolism has already been elucidated, most of the data so far available have reported observed changes at the phylum level. Furthermore, the physiological contribution of Firmicutes in the development of the obese phenotype is still being debated. In turn, some studies have observed a positive correlation between ratios of Bacteroidetes to Firmicutes and plasma glucose concentration, but not with BMI, although this was expected (Larsen et al., 2010).

Different mechanisms have been proposed in the attempt to understand the impact of microbiota both in maintaining metabolic health and in the development of obesity and T2D. Essentially, the intestinal microbial variability has been hypothesized as an important factor in four different processes, namely: (i) the modulation of energy homeostasis by regulating the energy harvest from diet, fat storage, lipogenesis, and fatty acid oxidation (host energy metabolism; Tilg et al., 2009; Musso et al., 2010); (ii) the modulation of the gut barrier integrity by regulating the epithelial permeability, the intestinal motility and the transport of digestion products such as short-chain fatty acids, which are an energy source for colonocytes (Samuel et al., 2008); (iii) the regulation of gastrointestinal peptide hormone secretion, by suppressing the secretion of the lipoprotein lipase inhibitor (fasting-induced adipose factor), determining the release of fatty acids from circulating triglycerides and lipoproteins in muscle and adipose tissue and promoting fat mass accumulation (Bäckhed et al., 2007); and (iv) the modulation of the host inflammatory state by contributing to the systemic increase of lipopolysaccharide, which impairs insulin sensitivity (metabolic endotoxemia; reviewed in Bäckhed et al., 2007; Cani et al., 2007, 2012; Sun et al., 2010; Vrieze et al., 2010; Shen et al., 2013). Evidence of the role of gut microbiota in the preservation of metabolic health also comes from the effect of prebiotics, such as non-digestible carbohydrates, namely non-digestible ingredients that are fermented by specific beneficial bacterial strains, selectively promote the growth and/or activity (release of end-products of bacterial fermentation) of the gastrointestinal microbiota, affecting favorably the host health (Gibson et al., 2010). The intake of prebiotics has in fact been described to act on host endocrine secretion, improve gut barrier integrity by increasing the release of glucagon-like peptide-2 (Cani et al., 2012; Dewulf et al., 2013), stimulate postprandial release of peptides involved in energy homeostasis and/or pancreatic functions such as the anorexigenic glucagon-like peptide-1 and peptide YY, and the decrease of orexigenic peptides such as ghrelin in plasma which in turn modulates food intake (regulators of appetite) and energy expenditure across the entire gastrointestinal tract (Piche et al., 2003; Delzenne and Cani, 2011; reviewed in Vrieze et al., 2010). Furthermore, evidence suggests that the modulation of the host metabolic health by prebiotics intake can be mediated to specific fermentation products (i.e., short-chain fatty acids, predominantly acetate, propionate and butyrate) produced by cross-feeding between *Eubacterium rectale* and *Bifidobacterium thetaiotaomicron* (Venema, 2010); *Propionibacterium* sp. and *Bacteroides* sp. (Hosseini et al., 2011); *Faecalibacterium prausnitzii* and *Roseburia intestinalis/Eubacterium rectale* (Duncan et al., 2004; Venema, 2010) respectively.

## The Metabolomic Approach

Due to the species specificity of several enzymatic machineries, the gut microbial composition and activity are likely to be characterized by the profile of small metabolites produced in the intestinal lumen, eventually absorbed through the intestinal barrier and further biotransformed by the host. Consequently, the complexity of microbial–host exchanges may be reflected in the specific chemical signature of host circulating biofluids (Nicholson et al., 2012). Metabolomics has recently attracted attention as the most suitable -*omics* technology for investigating complex, polygenic and multifactorial diseases with a strong metabolic etiology, such as obesity and T2D as well as the crosstalk of distinct predisposing factors in disease development and progression (Faber et al., 2007; Llorach et al., 2012; Du et al., 2013; Kurland et al., 2013). Aimed at the comprehensive analysis of the low- molecular- weight compounds contained in a biological system –by definition, metabolites comprise a plethora of primary or secondary derivatives of the intermediate metabolism (molecular weight below 900 and 2000 Dalton, depending on sources; Beckonert et al., 2010; Psychogios et al., 2011; Hadacek, 2015) metabolomics represents a powerful tool for exploring the crosstalk between the microbial and host metabolism in a more exhaustive fashion.

The workflow applied in metabolomic studies is broadly categorized into five main steps: (1) sample collection, (2) sample preparation, (3) data acquisition, (4) data analysis, and (5) biological interpretation of the results obtained (Llorach et al., 2012). The analytical techniques most commonly used for the characterization of the metabolome of a biological sample are MS and ^1^H-NMR. Both technologies have their advantages and disadvantages. ^1^H-NMR implies a non-destructive, non-selective, cost-effective, and relatively sensitive analysis while, compared to ^1^H-NMR, MS mainly offers potential advantages in terms of sensitivity and, if coupled to different separation techniques such as LC or GC, it provides a means of detecting a broader and complementary range of biomarkers (Faber et al., 2007). LC coupled to electrospray ionization MS is becoming the method of choice for the acquisition of profiling metabolites in complex biological samples (Scalbert et al., 2009) through both targeted (i.e., triple quadrupole-driven) and non-targeted (e.g., quadrupole time-of-flight-, linear trap quadrupole orbitrap-driven) approaches.

The present review aims to summarize the gut microbial–host cometabolites identified so far in humans in relation to obesity and/or T2D by targeted and untargeted metabolomic studies. Since the potential impact of some specific species in host metabolism has already been elucidated, an attempt to associate bacterial producers of the co-metabolites with the metabolic alterations related to the obese, diabetic, or diabese phenotype was also made. A critical view of the current limitations and future directions of metabolomics will accompany the discussion.

## Materials and Methods

### Search Strategy

The following keywords were searched for in the PubMed and Web of Science electronic databases: (Metabolom^∗^ [TW] or co-metabol^∗^ [TW] or host-gut metabo^∗^ [TW] or nuclear magnetic resonance [TW] or MS [TW] or magnetic resonance spectroscopy [TW]) AND (OBES^∗^ [TW] OR DIABET^∗^ [TW] OR DIABES^∗^ [TW]) AND (gut micro^∗^ [TW]). Species (human), language (English), and publication date restrictions (2000 to date, last search on November 27th, 2014) were imposed, but there were none for gender, age or ethnicity. Relevant references cited in the selected articles were additionally reviewed. Targeted and untargeted metabolomic approaches driven by ^1^H-NMR or MS techniques were both included in the selection. Low-molecular-weight (<1000 Da) metabolites significantly up- or downregulated in overweight and obese subjects with/without impaired glycemic control, with respect to controls (i.e., lean, healthy subjects), were the primary outcomes of interest of the review.

## Results And Discussion

### Characteristics of the Studies and Metabolic Variations

Only eight human studies successfully met the eligibility criteria for inclusion in the review (details in the Supplementary Material File). As summarized in **Table 1**, seven observational and one interventional study have so far applied a metabolomic approach and specifically identified changes in products of the gut microbial–host co-metabolism in overweight to obese individuals (BMI > 25 kg/m^2^) and/or several degrees of impaired glycemic control (ranging from IGT up to T2D) compared to control individuals. Other comorbidities were not described (i.e., hypertension, renal or liver dysfunction).

**Table 1:** Human metabolomic studies showing gut microbial–host co-metabolites significantly altered in obese and/or T2D diagnosed patients, respect to controls.

Observational Studies						

Disease	Participants^1^	Medication^2^	Approach (analytical technique)	Specimen	Changes respect to the CT group	Reference
Obesity + pre-T2D	Group 1 = 15 (0F) morbidly OB with IR	No	Non-targeted	Spot urine, fasting	↓Hippuric acid, *N-* methylnicotinate aaa 2-hydroxyisobutyrate	Calvani et al., 2010
	Group 2 (CT) = 10 (0F) healthy NW (with NGT)	No	(^1^H-NMR)			
Obesity + T2D (treated vs. not)	Group 1 = 15 (8F) OW with treated T2D	Metformin (15)	Non-targeted	Serum, fasting	aaaTrimethylamine-*N*-oxide	


	Group 2 (CT) = 20 (10F) OB with untreated T2D	No	(^1^H-NMR)			
Obesity + T2D (treated vs. not)	Group 1 = 20 (11F) OB with treated T2D	Glyburide (10), glimepiride (6), Gliclazide (4)	Targeted	Spot urine, fasting	↓Hippuric acid (untreated T2D) aaahippuric acid (with anti-T2D drugs)	Huo et al., 2015
	Group 2 = 20 (11F) OB with untreated T2D	No	(UPLC-MS)			
	Group 3 (CT) = 20 (10F) healthy OB (with NGT)	No				
Obesity + T2D	Group 1 = 30 (13F) OW to OB with untreated T2D	No	Non-targeted	Spot urine, fasting	↓Hippuric acid, *N-* methylnicotinate, ↓*N,N*-dimethylglycine, *N,N* dimethylamine	Salek et al., 2007
	Group 2 (CT) = 12 (4F) healthy NW to OW (with NGT)	No	(^1^H-NMR)			
Obesity + T2D	Group 1 = 40 (0F) OB with T2D	No (7), antidiabetic medication	Targeted	Serum, fasting	↓Cholate aaadeoxicholate ↓Gamma muricholate	Suhre et al., 2010
	Group 2 (CT) = 60 (0F) healthy OW (with NGT)		(UPLC-MS/MS)			
(pre-)T2D	Group 1 = 74 (42F) NW with T2D	No (48), metformin, acarbose, glipizide or repaglinide as a monotherapy (26)	Non-targeted	Serum, fasting	↓Choline (vs. NGT and vs. IGT)	Zhang et al., 2009
	Group 2 = 77 (44F) NW with IGT	No	(^1^H-NMR)			
	Group 3 (CT) = 80 (46F) healthy NW (with NGT)	No				
Obesity + pre-T2D	Group 1 = 12 (?F) OB with IGT	No	Non-targeted	Plasma, fasting	aaaGlycochenodeoxycholic acid	Zhao et al., 2010
	Group 2 (CT) = 39 (?F) healthy OB (with NGT)		(UPLC-qToF-MS)	Spot urine, fasting	↓Hippuric acid, 3-hydroxyhippuric acid, methyluric acid, methylxanthine	

**Single-arm intervention study (weight-loss plan with calorie restriction and exercise)**						

**Disease**	**Participants**	**Duration and associated clinical outcomes**	**Approach (analytical technique)**	**Specimen**	**Changes from baseline**	**Reference**

Obesity + pre-T2D	Group = 15 (15F) OB with IR (only 12 up to the end)	0, 14–17 weeks ↓BMI, body fat, V02, fasting	Non-targeted (GC-ToF-MS)	Plasma, fasting and 30, 60, 90, 120 min after OGTT	aaaTricarballylic acid (fasting and after OGTT)	Campbell et al., 2014


*^1^‘healthy’ stands for NGT subjects. Abbreviations: F, number of females in studies; CT, control; BMI, Body Mass Index; OW, overweight; OB, obese; NW, normal weight; NGT, normal glucose tolerance; IGT, impaired glucose tolerance; IR, insulin resistance; T2D, type 2 diabetes; pre-T2D, prediabetes;^1^H NMR, proton nuclear magnetic resonance; UPLC-qTOF-MS, reversed-phase ultra performance liquid chromatography coupled to electrospray ionization quadrupole time-of-flight mass spectrometry; UPLC-MS/MS, ultra-performance liquid chromatography tandem mass spectrometry; GC-TOF-MS, gas chromatography/time-of-flight mass spectrometry.*

*^2^Medication during the study.*

Overall, the study subjects, designs and objectives were quite heterogeneous despite the small number of retrieved studies (Supplementary Material File), thereby complicating an otherwise integrated and consistent picture of the metabolomic changes observed.

Urine (Salek et al., 2007; Calvani et al., 2010; Zhao et al., 2010; Huo et al., 2015), fasting serum (Huo et al., 2009; Zhang et al., 2009; Suhre et al., 2010) and plasma (Zhao et al., 2010; Campbell et al., 2014) were the biological samples used in these studies. A data-driven untargeted approach was chosen in the majority of the studies (Salek et al., 2007; Huo et al., 2009; Zhang et al., 2009; Calvani et al., 2010; Zhao et al., 2010; Campbell et al., 2014) while two of them provided quantitative information about known targeted metabolites (Suhre et al., 2010; Huo et al., 2015). The metabolic changes observed in these studies and the related interpretations are summarized in **Table 2.**

**Table 2:** Summary of the most significant gut microbial and host co-metabolites identified in the selected studies.

Class	Metabolite	Disease	Change^1^	Anti-T2D drugs effect	Sample	Interpretation	Reference	*Related bacteria (phyla)*
Bile acids (primary)	γ-muricholate (hyocholate) cholate glycochenodeoxycholate	Obesity + T2D	↓		Blood fluids	Bile acids are proposed as new metabolic integrators of whole body energy homeostasis that influence glucose and lipid metabolism. Subjects with diabetes exhibit alterations in the composition of the bile acid pool and their related biosynthetic pathway. A higher rate of conversion of primary to secondary bile acids by the gut microbiota has been implicated in the observed variation.	Suhre et al., 2010	Firmicutes^3^


		Obesity + T2D	↓		Blood fluids		Suhre et al., 2010	
		Obesity + pre-T2D	aaa		Blood fluids		Zhao et al., 2010	
Bile acids (secondary)	Deoxycholate	Obesity + T2D	aaa		Blood fluids		Suhre et al., 2010	
Vitamin metabolites	Choline	(pre-)T2D	↓↓^2^		Blood fluids	In the absence of anti-T2D treatment, alteration of choline metabolism (increased degradation) noticed in T2D patients may result from: (a) an altered demand, possibly by altered lipoprotein turnover/biosynthesis, (b) an altered gut microbiotal activity associated with T2D development, or (c) an osmotic compensation for raised blood glucose concentrations. Low levels of choline would also associate to the prevalence of OB/T2D complications, namely nonalcoholic fatty liver. When associated with metformin, may indicate a possible two-way relationship between the anti-T2D treatment and the gut microbiota. The intestinal bacteria composition would influence glucose metabolism and the mechanisms of action of metformin, and the drug would regulate back the gut microbial function.	Zhang et al., 2009	Firmicutes, Proteobacteria and Actinobacteria^4^


	TMAO	Obesity + T2D		aaa	Blood fluids		Huo et al., 2009	
	DMA, DMG	Obesity + T2D	aaa		Urine		Salek et al., 2007	
	*N*-methylnicotinate (trigonelline)	Obesity + (pre-)T2D	↓		Urine	Gut microbial class-specific product of the metabolism of niacin, which is an essential vitamin involved in major physiological functions such as coenzyme in tissue respiration, carbohydrate and lipid metabolism. Trigonelline regenerates glutathione stores that are depleted by oxidative stress in obesity. Moreover, low levels of trigonelline could suggest perturbation in nucleotide metabolism during T2D.	Salek et al., 2007; Calvani et al., 2010	NA

Organic acids and derivates	2-hydroxyisobutyric acid	Obesity + pre-T2D	aaa		Urine	Since their production is species specific at the colonic level, changes in their level may reflect (a) significant shifts in the subjects’ gut microbe ecology or functional differences in the microbiome metabolic activity between OB with IR and healthy lean individuals and (b) changes in the host metabolism/uptake of gut-derived metabolites, possibly related to a variation in the intestinal mucosa permeability after weight-loss plan with calorie restriction and exercise.	Calvani et al., 2010	Firmicutes^5^


	tricarballylic acid	Obesity + pre-T2D	aaa		Blood fluids		Campbell et al., 2014	
Phytochemical and purine metabolites	hippuric acid	Obesity + (pre-)T2D	↓↓↓↓	aaa	Urine	Changes in the production of hippurate and derivatives are generally connected to diet and gut microbial activities with the human metabolic phenotype and the blood pressure of individuals. They could indicate a relevant role of the gut microbiota in the pathogenesis of the pre-T2D state and could be related to age progression and gender effects on metabolism in T2D. The reversion of these changes by sulfonylurea treatment would confirm a beneficial effect of anti-T2D drugs on gut microbiota metabolism, besides glucose homeostasis. Higher concentrations in obese humans could reflect the known role of gut microbiota in energy metabolism and immune function of the host.	Salek et al., 2007; Calvani et al., 2010; Zhao et al., 2010; Huo et al., 2015	Firmicutes^5^


	3-Hippuric acid hydroxyhippuric acid				Urine		Zhao et al., 2010	
	Methyluric acid	Obesity + pre-T2D	↓		Urine	Gut microbiota-associated metabolite biomarkers, related to IGT. Accumulating evidence indicates that the gut microbiota is instrumental in the energy metabolism and immune function of the host.	Zhao et al., 2010	NA


	Methylxanthine	Obesity + pre-T2D	↓		Urine		Zhao et al., 2010	


*OB, obesity; T2D, type 2 diabetes; IGT, impaired glucose tolerance; NGT, normal glucose tolerance; SU, sulfonylurea; M, metformin hydrochloride; TMAO, trimethylamine-*N*-oxide; NMN, ox*N*-methylnicotinate; DMG, *N, N* dimethylglycine; DMA, dimethylamine. ^1^In respect to healthy controls. ^2^Also in T2D vs. IGT but not IGT vs. NGT. ^3^From: Begley et al., 2005; Ridlon et al., 2006; Fukiya et al., 2009; Labbé et al., 2014. ^4^From: Craciun and Balskus, 2012. ^5^From: Li et al., 2008.*

### Co-metabolism of Bile Acids

Two of the metabolomic studies described in this review highlighted a change in the circulating pool of BA in obese patients with insulin resistance or T2D, compared with BMI-matched healthy individuals (Suhre et al., 2010; Zhao et al., 2010). Alterations involved both human-derived (hepatic) structures (primary BA) and gut microbial-produced derivatives (secondary BA).

To the best of our knowledge, it is currently accepted that the bacterial enzymes involved in the biotransformation from primary to secondary BA are not shared across the whole microbial community, although they have been described so far in genera belonging to the four major phyla Firmicutes, Actinobacteria, Bacteroidetes and Proteobacteria (Labbé et al., 2014). Furthermore, according to Jones et al. (2014) Actinobacteria and Firmicutes clones would be the only ones able to degrade all conjugated BA, with Bacteroidetes species being limited to tauro- conjugation activities.

After their production in the liver and the eventual glyco- and tauro-conjugation (*N*-acyl amidation with glycine or taurine substituents), primary BA are secreted in the small intestine through the bilis (Ridlon et al., 2006; Hofmann and Hagey, 2008; Swann et al., 2011), where they are first subjected to deconjugation by a bacterial bile salt hydrolase enzyme produced by species of the four phyla, such as *Clostridium, Bacteroides, Lactobacillus, Bifidobacterium*, and *Enterococcus* (Begley et al., 2005). In the ileum, more than 95% of these BA undergo enterohepatic recycling (Swann et al., 2011; Kootte et al., 2012; Nicholson et al., 2012), are absorbed from the intestine and returned to the liver (Ridlon et al., 2006). The remaining 5% escape the enterohepatic circulation and reach the large bowel where the bioconversion into secondary BA is completed, especially by Firmicutes phyla *(Eubacterium* sp. and *Clostridium* sp.; Midtvedt, 1974; Nguyen and Bouscarel, 2008; Swann et al., 2011).

**FIGURE 1:**
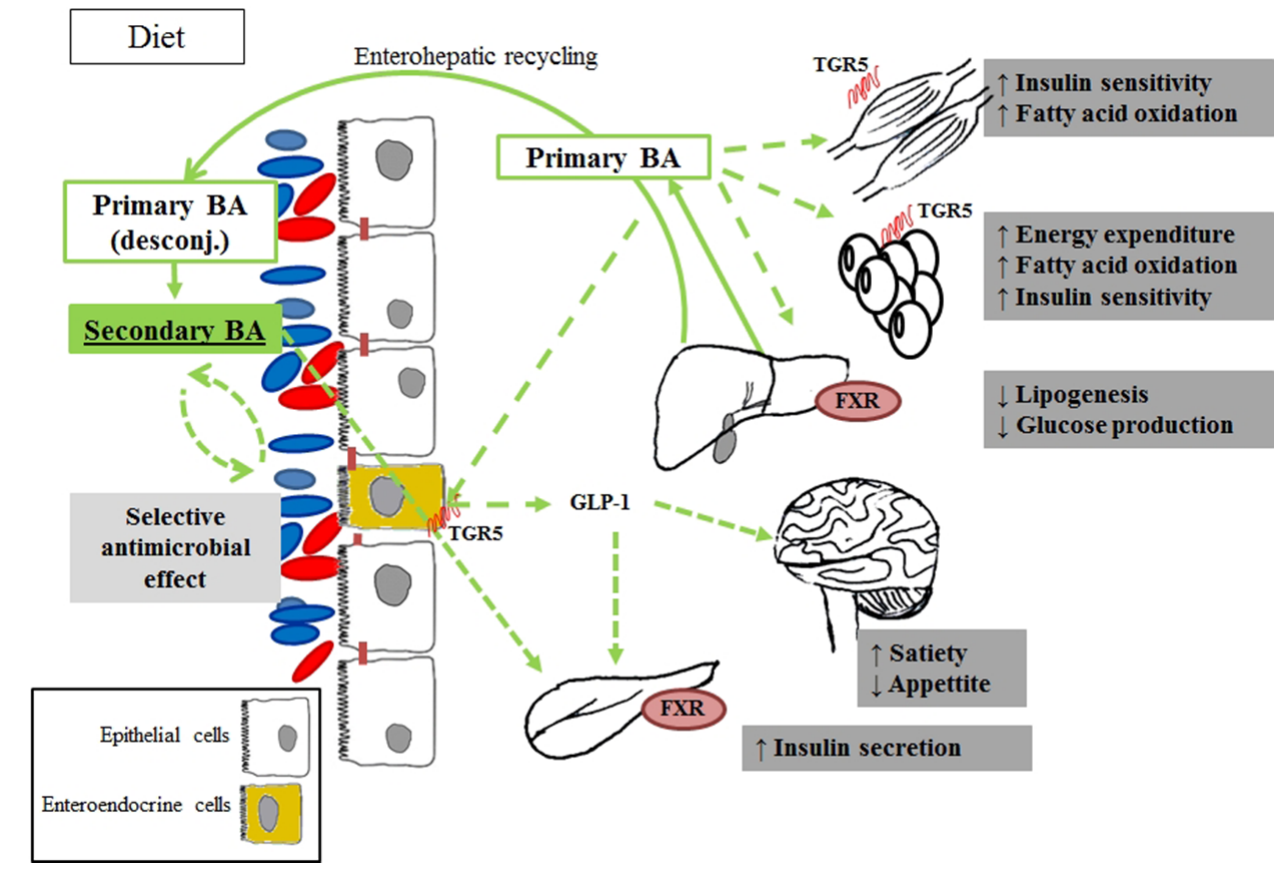
**Products of bile acids co-metabolism associated with obesity and diabetes via metabolomic studies.** After the ingestion of food, conjugated BA are secreted into the intestinal lumen, are subjected to deconjugation by the intestinal bacteria, and converted to secondary BA. The main effect of secondary BA is mediated by the FXR activation, which in turn act increasing insulin secretion and sensitivity in the pancreas. Primary BA is also involved in glucose metabolism by TGR-5 activation and glucagon-like peptide-1 (GLP-1) release. Host endogenous processes are in solid lines, while microbial production and metabolite actions are in dotted lines.

A decrease of primary BA and an increase of secondary BA was observed in the fasting serum of overweight patients with T2D, compared to healthy subjects (Suhre et al., 2010). The authors hence concluded that differences in the gut microbiota of diabetic patients may lead to higher rates of conversion from primary to secondary BA. In turn, Zhao et al. (2010) only observed an increase of glycochenodeoxycholic acid (primary BA) in the plasma of prediabetic individuals, while no changes were noticed in urine. Despite the only partial overlapping of the results, both studies suggested that overweight and obesity may not be the predominant factor implied in the metabolomic changes observed, and thus in linking impaired glucose homeostasis to alterations in BA pool composition. As argued in those studies, the variation of the BA pool in biofluids may depend on different factors, namely a change in the prevalence or activation of the gut microbial species implied in BA bioconversion or an altered reabsorption of BA through the gut mucosa, in turn produced by the disease itself, by dietary or other external changes (i.e., induced by bariatric surgery), or by a combination of these. In any case, the results indicated a probable implication of the modulation of BA biosynthetic pathways in the relationship between gut microbiota and insulin resistance (**Figure 1**).

These findings are in line with independent studies that recently associated changes in BA turnover with *diabesity*. In turn, a reduction of the bacterial enzymatic activities involved in the conversion of primary into secondary BA was observed in diabetic patients compared to healthy controls, and linked to Firmicutes phyla (Labbé et al., 2014). A very similar pattern was also reported in obese patients with diagnosed metabolic syndrome, treated with antibiotic agents (vancomycin and amoxicillin) and associated with both a decreased prevalence of the Firmicutes population and a reduction of peripheral insulin sensitivity (Vrieze et al., 2014). Taken together, these data suggest a possible link between BA and metabolic health (Lefebvre et al., 2009). In line with these findings, BA have recently been proposed as metabolic integrators of whole-body energy homeostasis implicated in the regulation of various metabolic pathways, including their own synthesis and enterohepatic circulation, triglyceride, glucose, and energy homeostasis, by acting as signaling molecules through receptor-dependent and -independent pathways. The role of a dysregulation of this BA-mediated metabolic control in the pathogenesis of T2D and co-morbidities, such as its attractiveness as a therapeutic target, is now beginning to be elucidated (reviewed in Prawitt et al., 2011). Their action on energy metabolism regulation would occur via both the activation of the nuclear receptor FXR and FXR-independent pathways, such as through the membrane receptor TGR5 expressed in several tissues including gall bladder, ileum, colon, and brown and white adipose tissue.

It is worth noting that preliminary evidence has shown that not all BA activate equally, and the microbial-derived production of secondary BA could be an important mechanism in the regulation of signaling pathways involved in the development of *diabesity* (Nguyen and Bouscarel, 2008). When gut microbial-derived secondary BA are bound to TGR5, the receptor is internalized and a series of adenylate cyclase-dependent signaling is triggered by activating distinct pathways involved in glucose and lipid energy metabolism (Kawamata et al., 2003; Thomas et al., 2008). In tissues, such as brown adipose tissue and muscle, this would lead to an increased mitochondrial activity and oxidative phosphorylation, which has been linked to an insulin sensitization both in genetic and diet-induced models of *diabesity* (Watanabe et al., 2006). In enteroendocrine L-cells (Kawamata et al., 2003), in turn, these signaling pathways would enhance glucose metabolism by stimulating the production of glucagon like peptide, thereby promoting insulin secretion. Finally, recent studies have also shown that BA secretion improves insulin secretion, insulin sensitivity and whole-body glucose homeostasis (reviewed in Thomas et al., 2008), improving liver and pancreatic function in obese mice (Thomas et al., 2009; Tremaroli and Bäckhed, 2012).

### Co-metabolism of Vitamins

Products of the gut microbiota-driven metabolic pathway of vitamins such as choline and niacin have been associated with obesity and diabetes.

#### Choline Metabolism

**FIGURE 2:**
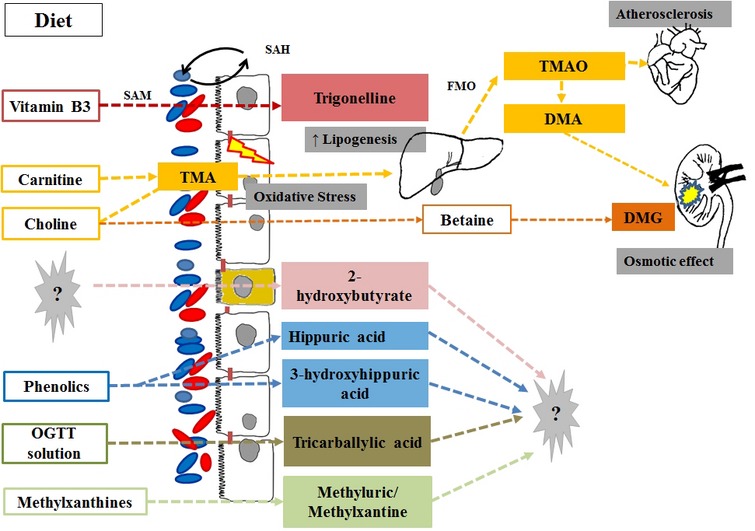
**Products of vitamin, phytochemical and purine co-metabolism associated with obesity and diabetes via metabolomics studies.** Intestinal microorganisms catalyze the conversion of dietary choline and carnitine into TMA with a direct effect on the intestinal mucose (increased oxidative stress) and is the substrate for the hepatic production of TMAO (associated with cardiovascular disease risk) and TMA. Choline may escape microbial degradation and convert into betaine and further products [i.e., dimethylglycine (DMG)] by mammalian mitochondrial pathways in the liver and kidney where they have an detrimental osmotic effects. For most of the host microbial co-metabolites associated with the diabese phenotype, the eventual role in glucose and lipid metabolism remains unknown. OGTT, Oral glucose tolerance test; SAH and SAM, *S*-adenosylmethionine to *S*-adenosylhomocysteine (methionine cycle).

Although humans may produce choline endogenously (*de novo* hepatic synthesis), dietary intake (e.g., from egg yolk, liver, muscle meats, fish, nuts, legumes) is necessary to meet the demand for body health maintenance (Blusztajn, 1998; Zeisel, 2000). Once dietary choline reaches the intestine, anaerobic intestinal microorganisms, mainly of Firmicutes and Proteobacteria phyla (Romano et al., 2015) catalyze its conversion to TMA, which may be further degraded to several methylamines by the gut microbiota (sym-xenobiotic pathway; Harris et al., 2012), or is absorbed and oxidized to TMAO by the hepatic FMO3 enzyme. Choline may also be converted into betaine and further products (e.g., dimethylglycine) by mammalian mitochondrial pathways in the liver and kidney (Lever and Slow, 2010) (**Figure 2**). The bacterial gene clusters responsible for anaerobic choline degradation started to be identified only recently. Bacterial species belonging to Firmicutes, Actinobacteria and Proteobacteria phyla have been revealed as possessing the necessary enzymatic activities, while Bacteroidetes would be apparently deprived (Craciun and Balskus, 2012). However, the complete diversity of species that contribute to TMAO production in humans still remains unknown (Romano et al., 2015).

Recent studies have shown that circulating levels of choline and TMAO are related to cardiovascular disease risk (Dumas et al., 2006; Micha et al., 2010; Wang et al., 2011; Koeth et al., 2013; Warrier et al., 2015), and the gut microbiota-driven TMA-FMO3-TMAO pathway has been particularly recognized as a key regulator of lipid metabolism and inflammation. Increased levels of TMAO have been observed in a leptin-deficient murine model of obesity and T2D (Gipson et al., 2008; Won et al., 2013) and revealed a contribution of gut microbiota to fatty liver phenotype in insulin-resistant mice (Dumas et al., 2006). The systemic perturbations of key metabolites of choline have also been related to the progression of T2D, suggesting that in the early stages of diabetes an attenuated conversion of choline into dimethylglycine may occur, which can be observed by the increased levels of TMAO and TMA, with a reversion of this behavior at a later stage of the disease (Guan et al., 2013).

Messana et al. (1998) published the first study linking TMAO and T2D in humans. Using a^1^H-NMR approach, increased levels of TMAO and dimethylamine were observed in the urine of diabetic individuals compared to a group of healthy individuals, and were present in high concentrations even in diabetics with good metabolic control (i.e., absence of glycosuria and glycohemoglobin). In the last decade, little progress has been made on the mechanisms that would directly or indirectly involve TMAO in the development of diabetes. Nevertheless, the potential role of an altered composition of the microbiota and its ability to metabolize choline in glucose homeostasis and disease development has become increasingly relevant (Dumas et al., 2006).

To the best of our knowledge, three further metabolomic studies observed a change in choline metabolism, which was associated with impaired glycemic control in humans, within a wide range of BMI (Salek et al., 2007; Huo et al., 2009; Zhang et al., 2009; **Table 1**). In all of them, fasting biosamples were analyzed, thereby avoiding fluctuations in choline concentrations due to dietary intake. Salek et al. (2007) carried out a ^1^H-NMR-driven metabolomic comparison of urinary changes linked to T2D both in animals (obese mice and rats with autosomal recessive defects in the leptin receptor gene – *db/db* and Zucker *fa/fa*, respectively), and individuals who were overweight to obsese (BMI = 25–40) compared to healthy lean controls. An increased excretion of a product of choline biotransformation, namely *N,N*-dimethylglycine and *N,N*-dimethylamine, distinguished the urinary metabolome of T2D in all species in the study (Salek et al., 2007), suggesting a possible increase in the choline turnover. The authors assumed that the *diabese* phenotype may have a major demand for choline, possibly due to an altered biosynthesis of lipoproteins, an altered metabolism of methylamines – which would play an important osmoregulatory role by degrading dietary choline – or to an altered intestinal microbiota composition (Salek et al., 2007). Although there is a scarcity of data in this regard, Firmicutes and Proteobacteria seem to be the most implicated phyla in the conversion of choline to TMAO (Romano et al., 2015). For this reason, the decline of choline circulating levels and increase of choline subproducts such as TMAO and DMA in obese subjects would be in line with the increase of the Firmicutes phylum associated with obese phenotype (Ley et al., 2006).

The role of BMI in the observed association was partly downsized in a second study (Zhang et al., 2009). By applying a similar untargeted and ^1^HNMR-driven approach, in fact, Zhang et al. (2009) demonstrated a decrease in the serum levels of choline in normal-weight subjects with T2D (BMI = 25.9 ± 9.0) compared to non-diabetic lean individuals (normal or IGT); however, no changes in the downstream products of choline metabolism were detected. Aside from increased insulin resistance, the decrease in serum choline is linked to a specific shift in the gut microbial community in the diabetic patients (relative increase in Firmicutes) and to an increase in the prevalence of T2D complications, as NAFLD (Zhang et al., 2009). The role of the microbial community hosted by *diabese* subjects in altering choline metabolism was also tested by assessing the effects of antidiabetic medication (Huo et al., 2009). As shown in **Table 1**, Huo et al. (2015) observed increased serum levels of TMAO in overweight diabetic subjects receiving metformin treatment *versus* untreated *diabese* controls, which may indicate an intestinal bacterial regulation function of metformin. It has already been suggested that antidiabetic treatments have a beneficial effect on gut microbiota metabolism (Huo et al., 2015), although the exact mechanisms are still unclear (Moreno-Navarrete et al., 2012). The authors suggested a link between the deregulation of choline metabolism in T2D and a rupture of the intestinal barrier by oxidative stress (Wei et al., 2008). In any case, a possible two-way relationship between anti-T2D treatment and gut microbiota has been hypothesized.

#### Niacin Metabolism

Alterations in the niacin (vitamin B3) metabolism have also been observed in association with obesity and T2D, and due to the overlapping in the choline/niacin catabolic pathways (i.e., via betaine and glycine metabolism), may also reflect a dysregulation in choline metabolism (Huang et al., 2012). Through a LC–MS-driven metabolomic approach, an altered urinary excretion of nicotinuric acid (*N*-nicotinoyl-glycine) was recently proposed as a potential marker of metabolic syndrome diagnostic traits and of cardiometabolic risk (Huang et al., 2012). Similarly, an association between the presence of trigonelline (betaine nicotinate) and obese and diabetic phenotypes has been proposed. Despite having a possible exogenous (dietary) origin, trigonelline is mostly biosynthesized by the gut microbiota during the conversion of *S*-adenosylmethionine to *S*-adenosylhomocysteine (methionine cycle). By applying a ^1^H-NMR-based metabolomics approach, Salek et al. (2007) found lower levels of trigonelline in the urine of diabetic (*db/db*) mice and humans, associated with a change in energy and tryptophan metabolism. Further animal (Salek et al., 2007; Won et al., 2013) and human studies (Calvani et al., 2010) confirmed a decline of trigonelline in obesity and diabetes, some authors suggesting that oxidative stress possibly has a role (i.e., via glutathione store depletion) in the observed relationship (Calvani et al., 2010). Trigonelline is known to be involved in major physiological functions including lipid and carbohydrate metabolism.

### Co-metabolism of Organic Acids and Derivates

Calvani et al. (2010) identified high levels of 2-hydroxyisobutyrate in the urine of obese people, and the change was associated with a reduced bacterial diversity in ‘obese’ gut microbiota possibly involved in nutrient and energy harvest (**Tables 1** and **2**). In particular, 2-hydroxyisobutyrate is a product of the microbial degradation of dietary proteins that escape digestion in the upper gastrointestinal tract, and its production has been associated with the presence of specific microbial members such as *Faecalibacterium prausnitzii* (Li et al., 2008), butyrate-producer species with anti-inflammatory effect and to be in low levels in obese and *diabese* individuals compared to healthy subjects (Qin et al., 2012). In addition, Campbell et al. (2014) observed that obese subjects involved in a dietary weight loss program had higher levels of tricarballylic acid after an OGTT compared with the fasting concentrations. Interestingly, tricarballylic acid is a product of gut microbial metabolism of food-derived trans-aconitate, described as an additive contained in the OGTT solution. Once again, the authors suggested a two-way relationship between the obese and gut microbial phenotype (tricarballylic acid production would in turn increase the metabolic activity of specific gut microbes associated with its production).

### Co-metabolism of Phytochemicals and Purines

Hippuric acid and 3-hydroxyhippuric acid are two normal urinary components mainly derived from the degradation of plant (poly)phenols and aromatic amino acids (i.e., phenylalanine and tryptophan) by a range of gut microbes, recently found to belong to *Clostridium* sp. (Li et al., 2008). The resulting benzoic acid is then absorbed, subjected to glycine conjugation reaction (by mitochondrial glycine *N*-acyltransferase) and finally excreted in urine (Huo et al., 2015). Decreased levels of hippuric acid (Salek et al., 2007; Calvani et al., 2010; Zhao et al., 2010) and 3-hydroxyhippuric acid in urine have been related to IGT and obesity (Zhao et al., 2010) in both animal and human studies. In turn, the downregulation was reduced in T2D patients after the treatment with sulfonylurea, suggesting the drug potentially has a protector effect on gut microbiota metabolism (Huo et al., 2015). However, a strict dietary assessment is mandatory to dismiss any diet-dependent variation among groups, due to the wide range of phenolic compounds leading to these last-step metabolites following microbial and human biotransformations (lack of specificity; Salek et al., 2007). Moreover, the reasons for their putative associations with obesity and T2D are unknown. Salek et al. (2007) suggested that hippurate could be related to age progression and gender effects on metabolism in T2D, but these suppositions need to be further investigated.

Zhao et al. (2010) observed that subjects with IGT had a reduced excretion of methyluric acid and methylxanthine, which are products of the microbial metabolism of methylxanthines contained for instance in coffee and tea. The authors tentatively interpreted the observed changes as the result of an altered gut microbiota in the presence of insulin resistance, although their putative role in glucose metabolism is still unknown.

## Conclusion

Current public health burdens such as obesity and T2D are complex, polygenic, multifactorial diseases with a strong metabolic etiology. Gut microbiota have recently been proposed as a crucial environmental factor in their development, but the metabolic complexity of the symbiotic interaction between the host individual and its microbial community, as well as the impact of this crosstalk between body weight changes and glucose homeostasis, are still unclear.

However, our review highlighted how few metabolomic studies have been specifically conducted so far to identify the role of the gut microbiota in the development and progression of obesity and T2D, at least in humans.

Despite the scarcity, heterogeneity and intrinsic limitations of the metabolomic studies conducted so far aimed at identifying the role of the gut microbiota in the development and progression of obesity and T2D (i.e., wide range of BMI and/or glycemic status evaluated, important sources of variability not considered including ethnic, gender effects, and dietary assessments), the results obtained by these data-driven metabolomic approaches are in line with findings independently obtained from *in vitro* or animal model studies. Products of the microbial/host metabolism of BA, vitamins (choline, niacin), branched fatty acids, purines and phenolic compounds have been described as being altered in (pre-)diabetic subjects, with or without increased BMI, compared with healthy controls. Moreover, few articles show a clear relation between metabolites and their bacterial producers in terms of the complexity of the gut microbiota and the cross-feeding mechanisms that would have combined bacterial effects in the colon ecosystem.

More efforts should be directed in the future toward expanding our knowledge of the metabolic interactions of the host and the gut microbiota, particularly through a strict evaluation of the lifestyle factors (i.e., diet) strongly involved in the modulation of this crosstalk.

## Conflict of Interest Statement

The authors declare that the research was conducted in the absence of any commercial or financial relationships that could be construed as a potential conflict of interest.

## ABBREVIATIONS

^1^H-NMR: proton nuclear magnetic resonance
BA: bile acids
IGT: impaired glucose tolerance
FMO3: flavin monooxygenase 3
FXR: Farnesoid X Receptor
GC: gas chromatography
LC: liquid chromatography
MS: mass spectrometry
NAFLD: non-alcoholic fatty liver disease
OGTT: oral glucose tolerance test
T2D: type 2 diabetes
TGR-5: *G*-protein coupled receptor
TMA: trimethylamine
TMAO: trimethylamine *N*-oxide.
