# Supplementary material for: Metabolomic insights into the intricate gut microbial–host interaction in the development of obesity and type 2 diabetes
Source: Front Microbiol. 2015 Oct 27;6:1151. doi: 10.3389/fmicb.2015.01151 (PMC4621279; doi:10.3389/fmicb.2015.01151)
Supplement: Supplementary file 1 [file Data_Sheet_1.PDF]

Supplementary Material

Metabolomic insights into the intricate gut microbial–host interaction in the development of obesity and type 2 diabetes

Magali Palau-Rodriguez[1a], Sara Tulipani[1,2a*], Maria Isabel Queipo Ortuño[2], Mireia Urpi-Sarda[1], Francisco J Tinahones[2,3], Cristina Andrés-Lacueva[1*]

[1] Biomarkers & Nutrimetabolomic Lab., Nutrition and Food Science Department, XaRTA, INSA, Campus Torribera, Pharmacy Faculty, University of Barcelona, Spain

[2] Biomedical Research Institute (IBIMA), Service of Endocrinology and Nutrition, Malaga Hospital Complex (Virgen de la Victoria), Campus de Teatinos s/n, University of Malaga, Malaga, Spain

[3] CIBER Fisiopatología de la Obesidad y Nutrición (CIBERobn), Instituto de Salud Carlos III (ISCIII), Madrid, Spain

[a] These authors contributed equally to the work.

***Correspondence:** Dr Sara Tulipani, Department of Nutrition and Food Science, XaRTA, INSA, Campus Torribera, Pharmacy School, University of Barcelona, Barcelona, Spain Fax:+34-934035931 E-mail: sara.tulipani@ub.edu; Dr. Cristina Andres-Lacueva, Biomarkers & Nutrimetabolomic Lab., Nutrition and Food Science Department, Campus Torribera, Pharmacy Faculty, University of Barcelona, Av. Joan XXIII s/n, 08028 Barcelona, Spain E-mail: candres@ub.edu Fax: +34-93-403-5931

1 Supplementary Data

1.1 Descriptive Results

The search in PubMed and Web of Science databases provided 75 and 162 publications respectively. After adjusting for duplicates, 170 remained, while the first-step selection based on a review of titles, abstracts and keywords excluded 121 publications.

The subjects in study were all adults (18–79 years old) but from different ethnic groups, including Han Chineses (Zhang et al., 2009), Caucasians (Campbell et al., 2014), black people (Campbell et al., 2014), Asians (Campbell et al., 2014), Hispanics (Campbell et al., 2014) and individuals of unknown ethnicity (Zhao et al., 2010; Calvani et al., 2010; Huo et al., 2009; Salek et al., 2007; Huo et al., 2014).

The objectives and study designs were quite heterogeneous, and ranged from exploring metabolomic difference between subjects with different metabolic status (Suhre et al., 2010; Zhao et al., 2010; Zhang et al., 2009; Calvani et al., 2010; Salek et al., 2007; Campbell et al., 2014) to assessing the effects of antidiabetic drugs on the gut microbial ecology and metabolic activity (Huo et al., 2009, 2014). Except for one case (Zhang et al., 2009), the reviewed studies were focused on the co-presence of both metabolic diseases (*diabesity*), and one prospective study described the gut microbial-related metabolomic changes following a diet and exercise weight-loss program in obese and insulin resistant females, thus showing a possible association with reduction in BMI (Campbell et al., 2014).
